# POEMS syndrome misdiagnosed as liver cirrhosis: a case report

**DOI:** 10.3389/fmed.2026.1815406

**Published:** 2026-04-22

**Authors:** Jiajing Xing, Weiwei Niu, Zhixin Feng, Hong Zhang, Shuohui Li, Fanhao Lu, Feng Gao, Limin Shi, Na Wang

**Affiliations:** Hebei Key Laboratory of Gastroenterology, Hebei Institute of Gastroenterology, Hebei Clinical Research Center for Digestive Diseases, Department of Gastroenterology, The Second Hospital of Hebei Medical University, Shijiazhuang, Hebei, China

**Keywords:** case report, diagnostic errors, liver cirrhosis, POEMS syndrome, vascular endothelial growth factor

## Abstract

POEMS syndrome is a rare multisystem paraneoplastic disorder characterized by polyneuropathy and monoclonal plasma cell proliferation, often leading to delayed diagnoses due to variable clinical manifestations. A 62-year-old woman presented with persistent bilateral eyelid edema as the initial symptom and abdominal distension as the predominant complaint. She was misdiagnosed with liver cirrhosis and remained undiagnosed for 6 years. Multidisciplinary evaluation revealed monoclonal IgA *λ* protein, markedly elevated vascular endothelial growth factor, and neurophysiological evidence of peripheral neuropathy, consistent with POEMS syndrome. Treatment with lenalidomide and dexamethasone, along with supportive care, led to improvement in abdominal distension and bilateral lower limb edema. This case underscores the need to consider POEMS syndrome in patients with unexplained eyelid edema accompanied by multisystemic manifestations. Given its potential to mimic other conditions, such as cirrhosis—as highlighted by this patient’s prolonged misdiagnosis—prompt and accurate recognition is essential to guide appropriate management, prevent irreversible organ damage, and improve long-term outcomes.

## Introduction

POEMS syndrome (polyneuropathy, organomegaly, endocrinopathy, monoclonal plasma cell disorder, and skin changes) is a rare paraneoplastic disorder caused by clonal plasma cell proliferation (1–3). Characterized by an insidious onset and marked clinical heterogeneity involving multiple organ systems, this condition often eludes timely diagnosis. The average time to definitive diagnosis typically ranges from 15 to 18 months (4), with initial misdiagnosis reported in up to 85% of cases (5).

Although existing literature on POEMS syndrome predominantly focuses on neurological damage and ascites (6, 7), this report presents an exceptionally rare case in which bilateral eyelid edema served as the initial manifestation. The patient was misdiagnosed with liver cirrhosis for many years primarily due to the presence of abdominal distension and ascites, which are typical features of cirrhosis. This case underscores the importance of considering POEMS syndrome in the differential diagnosis when such common manifestations are accompanied by unusual systemic findings.

## Case presentation

### Chief complaints

A 62-year-old woman, presenting with persistent edema of the eyelids for 6 years and abdominal distension for over 2 years, was admitted to our department in August 2025.

### History of present illness

The patient initially developed persistent bilateral eyelid edema and painless lymphadenopathy in the cervical and axillary regions 6 years prior, but did not seek medical attention at that time. Approximately 2 years ago, she began to experience proximal myalgia, most pronounced in the lumbosacral and gluteal regions, followed by recurrent, persistent, symmetrical pitting edema of both lower limbs and persistent abdominal distension. Long-term administration of diuretics failed to relieve these symptoms. Approximately 1 year before admission, she developed intermittent low-grade fever, peaking at 37.5 °C in the mornings and typically resolving by evening, accompanied by limb weakness. She was evaluated at several hospitals. Given her 20-year history of daily self-medication with two packets of Compound Paracetamol, Amantadine and Chlorphenamine Granules for intermittent headaches, a preliminary diagnosis of monoclonal gammopathy and liver cirrhosis was considered. She received diuretics and immunomodulatory agents, but her response was suboptimal. Abdominal computed tomography (CT) revealed imaging features suggestive of cirrhosis, splenomegaly, portal hypertension, lymphadenopathy, ascites, and subcutaneous and pericardial effusion. Liver stiffness was measured using shear wave elastography (SWE) with an Aixplorer^®^ device (SuperSonic Imagine, Aix-en-Provence, France). The examination was performed with the patient in the supine position, yielding three valid measurements of 14.7, 15.0, and 15.5 kPa, with a median liver stiffness measurement (LSM) of 15.1 kPa. According to the manufacturer’s reference, this value corresponds to Metavir F4 fibrosis. Of note, this measurement was obtained in the presence of moderate ascites, which may confound the accuracy of elastography; thus, the value should be interpreted with caution. Lymph node biopsy demonstrated reactive lymphoid hyperplasia. Bone marrow examination revealed scattered granulocytic and erythroid cells, absent megakaryocytes, and approximately 1% interstitial plasma cell infiltration without amyloid deposition. Flow cytometry and fluorescence *in situ* hybridization (FISH) revealed no clonal plasma cell population. Genetic analysis identified an ATR gene variant. Chest CT demonstrated chest wall edema. Upper gastrointestinal endoscopy revealed chronic atrophic gastritis (C2), while colonoscopy findings were unremarkable.

Hematological analysis showed decreased white blood cell and red blood cell counts, with a normal platelet count. Serum biochemistry demonstrated normal liver enzymes and bilirubin; however, it revealed decreased cholinesterase levels and elevated serum creatinine (124 μmol/L, previously normal) and uric acid (433 μmol/L). Coagulation testing indicated a prolonged prothrombin time. Immunological workup revealed an antinuclear antibody (ANA) titer of 1:320, anti-double-stranded DNA antibody at 41.1 IU/mL, and positivity for perinuclear antineutrophil cytoplasmic antibody (P-ANCA). Anti-thyroglobulin antibody was markedly elevated (>1,000 IU/mL, previously 183.9 IU/mL). Serum procalcitonin was 0.46 ng/mL. Serum immunofixation electrophoresis identified an IgA *λ* monoclonal (M) protein, and free light chain assay showed a serum free kappa of 36.3 mg/L and λ of 312 mg/L. Serum protein electrophoresis demonstrated an elevated *α*-1 globulin fraction (5.4%). Infectious disease screening was negative for tuberculosis (T-SPOT. TB, TB-IgG), hepatitis viruses, and HIV. A panel of four tumor markers was also negative.

**Figure 1:**
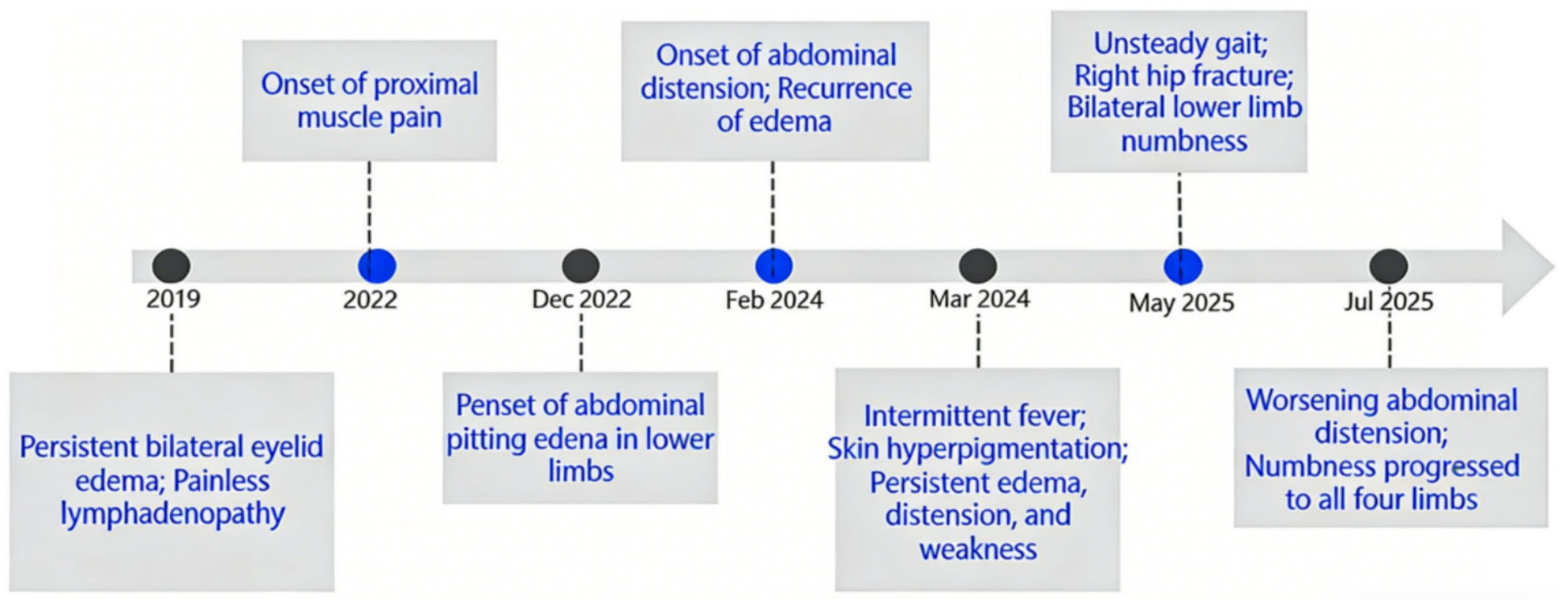
Clinical timeline of symptom progression in the present case.

Nine months before admission, her body temperature returned to normal; however, abdominal distension, bilateral eyelid and lower limb edema, and limb weakness persisted. During this period, she developed skin hyperpigmentation and leukonychia. Five months before admission, her proximal myalgia worsened, leading to gait instability and a right hip fracture. One month before admission, her abdominal distension intensified, accompanied by new-onset limb numbness. The timeline of symptoms is presented in Figure 1.

### History of past illness

Her medical history included hypertension, type 2 diabetes mellitus, an old cerebral infarction, and cervical spondylosis. She had experienced intermittent precordial discomfort for over 20 years and recurrent headaches for which she had self-administered an over-the-counter herbal cold remedy daily for over two decades, discontinuing it 2 months ago.

### Personal and family history

The patient denied any family history of malignant tumors.

### Physical examination

**Figure 2:**
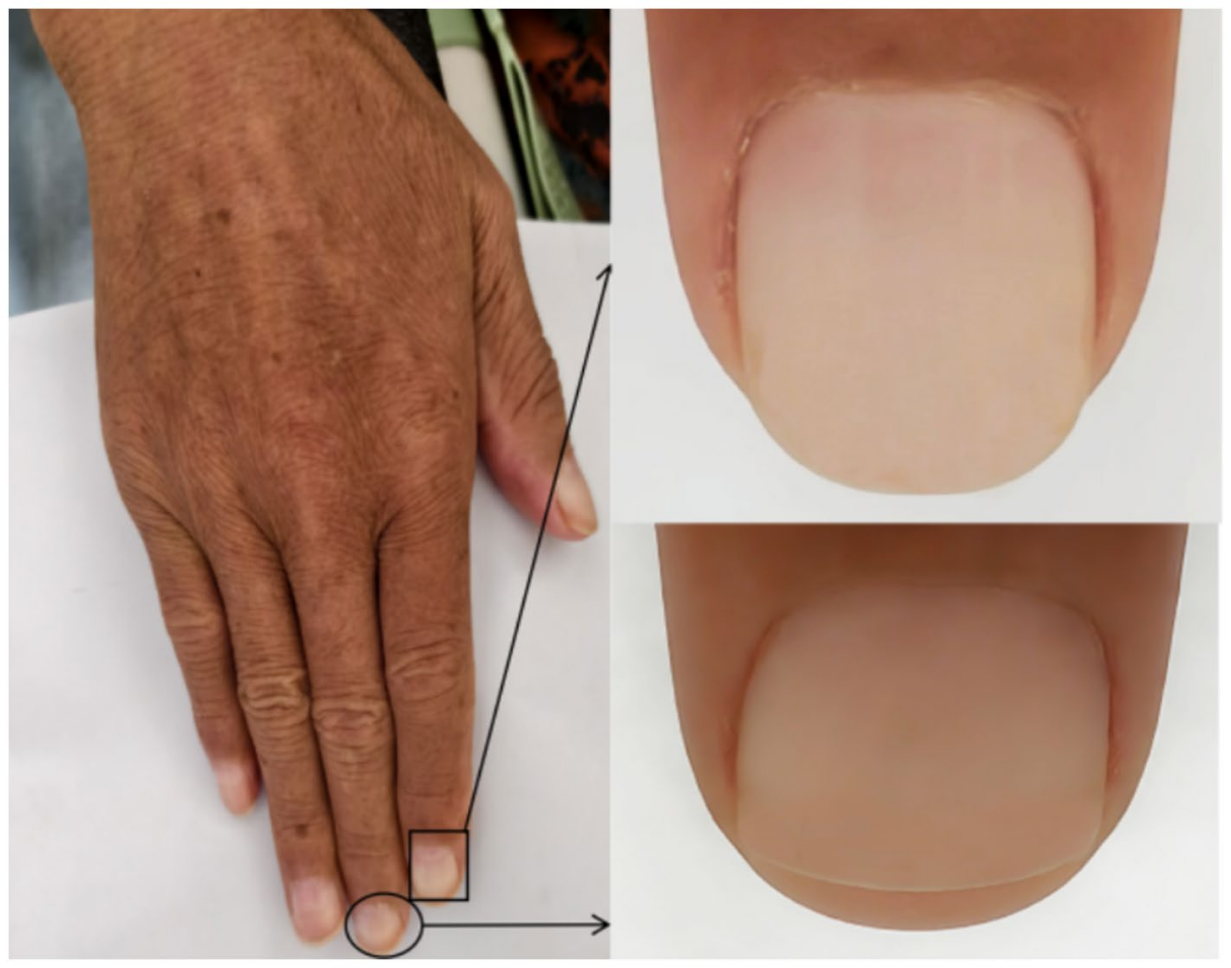
Clinical photograph showing leukonychia involving multiple fingernails.

On physical examination, cutaneous hyperpigmentation and leukonychia (Figure 2) were noted. Palpable lymphadenopathy was observed in the axillary and inguinal regions. The abdomen was distended, with positive shifting dullness; the liver and spleen were palpable below the costal margins. Muscle strength in the lower limbs was grade IV, accompanied by pitting edema.

### Laboratory and imaging examinations

**Figure 3:**
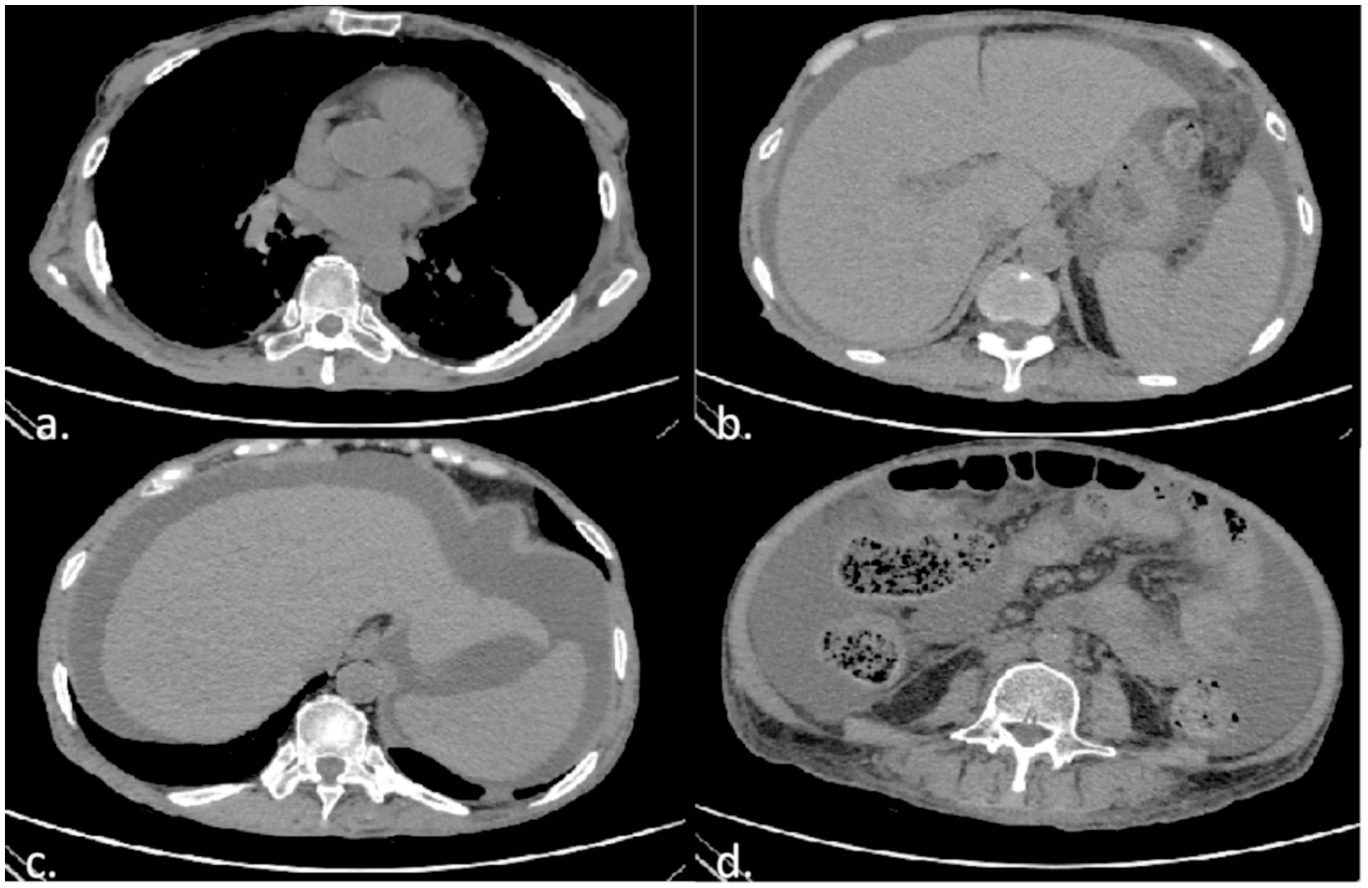
CT imaging of the chest, abdomen, and pelvis demonstrating multisystem manifestations. **(a)** Chest CT demonstrates pericardial effusion. **(b)** Abdominal CT shows an irregular, wavy liver contour and splenomegaly. **(c)** Abdominal CT reveals free intraperitoneal fluid accumulation. **(d)** Pelvic CT shows thickened, edematous intestinal walls and subcutaneous edema.

Following admission in August 2025, a comprehensive neurological and systemic evaluation was performed. Nerve conduction studies (NCS) indicated generalized peripheral neuropathy, characterized by absent waveforms in the motor and sensory nerves of the lower limbs, prolonged latency, reduced amplitude, and decreased conduction velocity in both motor and sensory nerves of the upper limbs. CT of the abdomen and chest revealed pleural and pericardial effusions, radiological features consistent with cirrhosis, splenomegaly, portal hypertension, subcutaneous edema of the abdominal and pelvic walls, and thickened, edematous intestinal walls (Figure 3). Serum albumin: 29.5 g/L. Serum immunofixation electrophoresis identified IgA and *λ* light chains (M-protein). Serum protein electrophoresis showed an α1-globulin fraction of 5% and an M-protein concentration of 0.7%, while urine protein electrophoresis was negative. The vascular endothelial growth factor (VEGF) level was markedly elevated at 226.69 pg./mL. Endocrine assessment revealed an anti-thyroglobulin antibody level of 23.9 IU/mL, serum prolactin at 26.89 ng/mL, elevated TSH (10.65 μIU/mL) with low FT4 (6.91 pmol/L) and FT3 (3.5 pmol/L), consistent with hypothyroidism. Vitamin D levels were low at 6.7 ng/mL. Ascites fluid analysis revealed a serum-ascites albumin gradient (SAAG) of 12.8 g/L (1.28 g/dL). Autoimmune serology showed a maximum ANA titer of 1:320, positive P-ANCA; additionally, objective tests revealed reduced salivary secretion (0.4 mL/15 min, normal >1.5 mL), bilateral Schirmer’s test results of 5 mm/5 min, and negative corneal staining. The direct antiglobulin test was positive. All tests for tuberculosis, viral infections, and common tumor markers were negative.

### Final diagnosis

A diagnosis of POEMS syndrome was established based on the major diagnostic criteria. These included peripheral neuropathy (manifesting as limb weakness and motor deficits), systemic edema (involving the eyelids, lower limbs, subcutaneous tissues of the abdominopelvic and chest walls, and intestinal wall, with accompanying pericardial and pleural effusions), organomegaly (splenomegaly), endocrinopathies (hypothyroidism, hypogonadism, and diabetes mellitus), and characteristic skin manifestations (hyperpigmentation and leukonychia). Key points regarding the differential diagnoses are presented in Table 1.

**Table 1:** Differential dIagnosis considered and the rationale for their exclusion.

Differential diagnosis	Overlapping features	Negating features
Cirrhosis	Hepatosplenomegaly, ascites, hepatic fibrosis	Normal liver injury markers (e.g., bilirubin, transaminases); presence of multisystem involvement (elevated VEGF, neuropathy) unexplained by cirrhosis alone; LSM reached F4 but was obtained in the presence of ascites, which may affect accuracy; multisystem involvement not explained by cirrhosis
Sjögren’s syndrome	Reduced salivary flow, abnormal Schirmer’s test, low-titer ANA and P-ANCA positivity	Absence of typical dry mouth/eye symptoms, negative corneal staining, repeatedly negative anti-SSA/SSB antibodies, and lack of confirmatory lip biopsy
Multiple myeloma	Fatigue, renal impairment	Bone marrow examination showed only approximately 1% plasma cells, below the 10% diagnostic threshold
Diabetic peripheral neuropathy	History of diabetes, electromyography indicating nerve conduction impairment	Disproportionately severe sensorimotor neuropathy, with involvement of both sensory and motor modalities, relative to the patient’s short diabetic history, whereas diabetic neuropathy predominantly affects sensory fibers

VEGF, Vascular endothelial growth factor; ANA, Antinuclear antibody; P-ANCA, Perinuclear antineutrophil cytoplasmic antibody; anti-SSA/SSB antibodies, Anti-Sjögren’s syndrome-related antigen A/B antibodies.

### Treatment

The patient was initiated on combination therapy with lenalidomide and dexamethasone, along with comprehensive supportive care. Treatment adherence was excellent throughout the clinical course.

### Outcome and follow-up

The patient reported marked improvement in abdominal distension and bilateral lower limb edema. Follow-up abdominal ultrasound at 3 months confirmed improvement, showing a reduction in pelvic free fluid from 5.1 to 2.7 cm. At 6 months, repeat abdominal ultrasound demonstrated complete resolution of ascites. She continues to be monitored regularly for disease progression through clinical evaluation and serial VEGF measurements (8).

## Discussion

POEMS syndrome is a rare paraneoplastic disorder characterized by abnormal plasma cell proliferation and elevated levels of inflammatory cytokines. The M-protein or lymphokines secreted by plasma cells exert toxic effects on peripheral nerves, endocrine glands, the reticuloendothelial system, and the hematopoietic system, resulting in multisystem involvement (9). Among these cytokines, VEGF has been most closely linked to disease activity, with elevated levels detected in over 85% of patients (10). A plasma or serum VEGF concentration of ≥200 pg./mL provides a specificity of 95% and a sensitivity of 68% for the diagnosis (11). Increased VEGF secretion promotes endothelial proliferation and enhances plasminogen activator activity, leading to angiogenesis and increased vascular permeability (12). This mechanism is believed to underlie the development of polyserous effusions in POEMS syndrome.

The global incidence of POEMS syndrome is approximately 0.3 per 100,000 population (10). Clinical manifestations demonstrate significant geographic and ethnic heterogeneity. In Asian cohorts, the Japanese nationwide survey reported a median age at onset of 54 years with a male-to-female ratio of 1.5:1 (10); the Chinese series of 99 patients showed a median age of 45 years, male-to-female ratio of 1.4:1, diagnostic delay of 18 months, and frequencies of peripheral edema (85%), hepatomegaly (47%), splenomegaly (71%), and ascites (55%) (4); the Korean multicenter study of 84 patients reported a median age of 53 years with 63.1% male (7). A shared characteristic among Asian populations is the significantly higher prevalence of extravascular volume overload compared with Western cohorts (China 88%, Japan 91% vs. United States 29%) (4). The Latin American GELAMM study (46 patients from 24 centers across 8 countries) demonstrated a median age of 52 years, male-to-female ratio of 1.9:1, and diagnostic delay of 7.7 months (13). Notably, organomegaly profiles differed between Chinese and Latin American patients: splenomegaly was observed in 71% of Chinese patients versus 17% in the Latin American cohort (13). This disparity may reflect variations in imaging protocols, diagnostic criteria, or disease spectrum across regions. The present patient from North China presented with eyelid edema as the initial manifestation and was misdiagnosed with liver cirrhosis for 6 years—a diagnostic delay exceeding that reported in Asian series, underscoring the need for heightened awareness of atypical phenotypes.

POEMS syndrome is characterized by highly heterogeneous clinical presentations, frequently resulting in delayed diagnosis. This case underscores two key diagnostic pitfalls: the failure to recognize atypical initial symptoms and insufficient exploration of edema pathogenesis, both contributing to prolonged misdiagnosis. Notably, the patient’s predominant and initial manifestation was persistent bilateral eyelid edema, rather than the classic peripheral neuropathy typical of POEMS syndrome. This uncommon presentation significantly delayed accurate diagnosis over years of medical evaluations. Although multiple hospitals conducted evaluations for cardiac, hepatic, and renal disorders, investigations into immune and hematologic causes were insufficient. The patient’s proximal muscle pain was also overlooked, contributing to the missed diagnosis. Eyelid edema as the first sign of POEMS syndrome is exceedingly rare and is likely associated with markedly elevated serum VEGF levels. The loose subcutaneous tissue and rich venous and lymphatic networks of the eyelids predispose them to fluid retention. Moreover, plasma cell infiltration involving the lacrimal glands or extraocular muscles may further impair local venous return, worsening edema. Therefore, unexplained bilateral eyelid edema should prompt consideration of multisystem disorders, including immune and hematologic etiologies.

Secondly, the conceptual framework guiding the differential diagnosis of edema was limited. Although hypothyroidism and hypoalbuminemia were present, the edema pattern was inconsistent: the patient had persistent pitting edema, contrasting with the non-pitting myxedema typical of hypothyroidism. A serum albumin level of 29.5 g/L usually correlates with mild peripheral edema but cannot explain the longstanding bilateral eyelid edema and polyserositis, suggesting a primary mechanism of increased capillary permeability rather than hypoalbuminemia alone. The significantly elevated serum VEGF supports a diagnosis of VEGF-driven microvascular leak syndrome in POEMS syndrome as the principal cause of the ascites.

A search of the PubMed database using the keyword “POEMS Syndrome” for case reports published over the past three decades shows that most reports focus on neurological manifestations, abdominal distension, or lower limb edema. In contrast, the presentation with bilateral eyelid edema as the initial manifestation in our case is extremely rare. No English-language case reports describing bilateral eyelid edema as the initial or early manifestation of POEMS syndrome were identified. Sporadic descriptions exist in the Chinese literature (a 36-year-old female who presented with bilateral eyelid edema for 2 years in 2002; a 55-year-old male with eyelid swelling for 10 months in 2008; and a patient in 2022 who developed eyelid and bilateral lower limb edema in the early disease stage). However, these have not gained widespread international attention. The uniqueness of the present case lies in the long-standing eyelid edema that was overlooked, together with concurrent ascites and signs of portal hypertension, leading to a misdiagnosis of cirrhosis for 6 years—a diagnostic delay far exceeding the median of 18 months reported in Asian cohorts. For future clinical practice, we propose that when encountering unexplained bilateral eyelid edema, especially when accompanied by generalized edema or signs of portal hypertension, clinicians should consider POEMS syndrome in the differential diagnosis and promptly perform screening tests including serum VEGF, immunofixation, and light chain quantification.

The patient’s long-term use of Compound Paracetamol, Amantadine and Chlorphenamine Granules (containing paracetamol, chlorphenamine maleate, caffeine, and artificial cow bezoar) likely contributed to liver injury through dual mechanisms. Chronic paracetamol metabolism can deplete glutathione, leading to the accumulation of the toxic metabolite NAPQI and direct hepatocyte damage (14). Additionally, chlorphenamine maleate may promote immune-mediated liver injury and disrupt immune homeostasis, collectively fostering a pro-fibrotic inflammatory microenvironment. However, the persistence and progression of systemic symptoms despite drug cessation were inconsistent with isolated drug-induced injury. Evidence suggests that elevated VEGF, a hallmark of POEMS syndrome, may contribute to hepatic fibrosis by upregulating procollagen synthesis in hepatic stellate cells (15) and amplifying the pro-fibrotic inflammatory milieu (16). Given the patient‘s multisystem involvement, POEMS syndrome is considered the primary cause of both the systemic manifestations and the hepatic fibrosis, while long-term use of hepatotoxic medications may have acted synergistically to promote fibrosis.

The patient’s clinical presentation—ascites and splenomegaly indicative of portal hypertension—closely mimicked decompensated cirrhosis. This impression was further supported by a 20-year history of paracetamol use, which provided a plausible etiology for drug-induced liver injury. Meanwhile, although shear wave elastography (SWE, Aixplorer^®^) measures liver stiffness using a technical principle different from that of vibration-controlled transient elastography (VCTE, FibroScan^®^), extensive studies have demonstrated high consistency between the two methods in staging liver fibrosis. SWE indicated increased liver stiffness, making it more likely to be misdiagnosed as liver cirrhosis. According to classical criteria, an elevated SAAG usually indicates portal hypertension. However, some experts suggest that when the SAAG is below 1.5 g/dL, it tends to favor increased capillary permeability due to VEGF overproduction. Our patient’s SAAG value of 1.28 g/dL fits this pattern. Since the report by Inoue et al., non-cirrhotic portal hypertension has been recognized as a rare but established manifestation of POEMS syndrome, with their autopsy case providing the histological evidence of idiopathic portal hypertension in the liver (17). In our patient, evidence of portal hypertension (splenomegaly, ascites) was present without severe laboratory signs of liver dysfunction (18), raising the possibility that non-cirrhotic portal hypertension may have played a greater role than fibrosis in the patient’s portal hypertensive features. Additionally, the presence of multisystem involvement further supports that the clinical presentation is more consistent with POEMS syndrome rather than meeting the diagnostic criteria for decompensated cirrhosis. In our patient, the liver stiffness measurement was performed in the presence of moderate ascites, which may confound the accuracy of elastography. Therefore, we suggest that in similar future cases, elastography should be repeated after adequate ascites drainage to obtain more reliable liver stiffness values. Unfortunately, repeat liver stiffness measurement was not performed after ascites resolution in this patient. Hence, in similar cases in the future, performing a liver biopsy to obtain histological evidence should be considered.

In the absence of liver biopsy, other causes of non-cirrhotic portal hypertension, such as nodular regenerative hyperplasia (NRH), cannot be completely excluded. However, abdominal ultrasound showed no focal nodular changes or “mottled” pattern characteristic of NRH, and the patient’s portal hypertension improved markedly after POEMS-directed therapy, whereas NRH as an independent organic lesion is not typically reversible. Therefore, although NRH cannot be entirely ruled out, its likelihood as an independent etiology is low.

Notably, POEMS syndrome often exhibits overlapping features with autoimmune disorders, complicating differentiation. In this case, findings such as reduced salivary flow, abnormal tear secretion, and low-titer ANA positivity raised the possibility of concurrent Sjögren’s syndrome (SS). However, the overall evidence does not support a separate SS diagnosis (19, 20). Current evidence indicates that VEGF-mediated periductal edema, microvascular proliferation, and mild inflammation can lead to glandular dysfunction (21) in POEMS syndrome, providing a unifying pathophysiology for both thyroid (22) and exocrine gland involvement. Thus, the observed glandular abnormalities and serological markers are more consistent with POEMS syndrome alone. Further investigations such as salivary gland ultrasound or lip biopsy may be considered for definitive exclusion of SS.

Most cases emphasize neurological involvement, abdominal distension, or lower limb edema as dominant presentations (23). To our knowledge, bilateral eyelid edema as the presenting symptom has rarely been documented, making the case particularly notable. The diagnostic process was further confounded by the patient’s history of chronic use of hepatotoxic medications and the presence of portal hypertension, both of which initially suggested liver cirrhosis. This case highlights the importance of considering multisystem disorders when evaluating atypical presentations and highlights the value of integrating hematological and immunological assessments early in the workup. By broadening diagnostic consideration, clinicians may achieve earlier detection and management of POEMS syndrome, thereby reducing irreversible organ damage and improving outcomes.

Current evidence suggests that the number of clinical features at diagnosis does not correlate with prognosis in POEMS syndrome (24). A risk stratification model developed by the Peking Union Medical College group in China incorporates age over 50 years, pleural effusion, pulmonary hypertension, and estimated glomerular filtration rate (eGFR) to guide clinical decision-making. Treatment strategies depend on transplant eligibility. Autologous stem cell transplantation is recommended as first-line therapy for intermediate- and high-risk patients (25), while lenalidomide-based regimens represent preferred alternatives for transplant-ineligible individuals (26, 27). Comprehensive supportive care remains essential for managing endocrine dysfunction, peripheral neuropathy, and systemic complications.

This case illustrates the value of a multi-system approach in diagnosing and managing POEMS syndrome. Prompt initiation of lenalidomide and dexamethasone therapy, coupled with supportive care and excellent patient adherence, resulted in significant symptomatic improvement. The main limitations of this case are threefold: irreversible organ damage resulting from the prolonged diagnostic delay; the lack of liver biopsy, which precluded histological confirmation; and the absence of repeat liver stiffness measurement after ascites resolution. Nonetheless, the diagnosis was firmly established based on fulfillment of the major diagnostic criteria for POEMS syndrome.

## Conclusion

POEMS syndrome should be highly suspected in patients presenting with polyneuropathy accompanied by polyserositis. The occurrence of unexplained, non-specific manifestations such as bilateral eyelid edema, abdominal distension, and cutaneous hyperpigmentation warrants prompt and comprehensive diagnostic evaluation. Early recognition and intervention are essential to prevent severe complications.

## Data Availability

The original contributions presented in the study are included in the article/supplementary material, further inquiries can be directed to the corresponding author.
